# EHDS2 pilot infrastructure for the secondary use of health data

**DOI:** 10.1093/eurpub/ckac129.368

**Published:** 2022-10-25

**Authors:** E Bacry, M Jendrossek

**Affiliations:** Health Data Hub, Paris, France

## Abstract

The EHDS2 Pilot consortium brings together 16 European partners (national platforms, research infrastructures, EU agencies and associations in the area of health) to respond to the EC's call for projects to set up a test version of the future European Health Data Space (EHDS) for the secondary use of health data (EHDS2). It aims to build this pilot version of EHDS2 by interconnecting data platforms in a network of nodes, implementing and testing a first user journey for creating, deploying and running health data research projects at EU level. The network will investigate the technical tools and set up the standards allowing researchers to query the metadata of all nodes, to request selected data via a single application form and to analyze data across national nodes. To build this network, the EHDS2 Pilot will propose legal and technical frameworks based on use cases selected by the EC. These will also serve to illustrate the powerful impact of exploiting health data from several countries and address topics from population health to healthcare pathways, cancer, rare diseases and genomics. This presentation will give an overview of the planned activities by the coordinating team.

